# Surgical and Non-surgical Interventions for Intermittent Exotropia: A Systematic Review of Randomized Controlled Trials

**DOI:** 10.7759/cureus.112659

**Published:** 2026-07-14

**Authors:** Abdulaziz Almadani, Mohammed N Almutairi, Elyas A Kirat

**Affiliations:** 1 General Medicine, King Saud Bin Abdulaziz University for Health Sciences, Riyadh, SAU; 2 General Medicine, Diriyah Hospital, Riyadh Third Health Cluster, Riyadh, SAU; 3 General Medicine, College of Medicine, King Saud Bin Abdulaziz University for Health Sciences, Riyadh, SAU

**Keywords:** intermittent exotropia, medial rectus resection, orthoptic therapy, over-minus lens therapy, patching, strabismus surgery

## Abstract

Intermittent exotropia is one of the most common forms of childhood-onset strabismus, characterized by an outward deviation of the eye that occurs intermittently. Treatment can be surgical, non-surgical, or a combination of both. Non-surgical treatments include occlusion therapy, the use of minus lenses to stimulate convergence, and orthoptic therapy consisting of fusion exercises. Surgery is recommended to normalize binocular function when the patient experiences an increased frequency of strabismus. Given the lack of a universal gold-standard treatment, the decision whether to perform surgery and the optimal timing of surgical intervention remain persistent challenges. This systematic review aims to assess and compare the effectiveness of surgical and non-surgical management of intermittent exotropia. It also aims to explore the factors associated with lower or higher success rates among different treatment approaches for intermittent exotropia. A systematic search of studies published in PubMed, Cochrane Library, and ScienceDirect was completed on the surgical and non-surgical management of intermittent exotropia. An investigator searched a set of medical databases for randomized controlled trials and controlled clinical trials in English published within the last 10 years to ensure relevance. Major outcomes of interest included motor alignment success and improvement in control of intermittent exotropia. Secondary outcomes included changes in angle of deviation, binocular function, recurrence rates, need for re-intervention, and treatment-related adverse events. The target population was intermittent exotropia patients who underwent surgical and non-surgical treatments. The quality of the studies included was evaluated using the Cochrane Risk of Bias 2 (RoB 2) tool for randomized trials. This systematic review comprised 13 studies, most of which were randomized controlled trials, with a small number of secondary analyses and post hoc analyses of randomized controlled trials. The majority of the research focused on pediatric populations; however, some included adult patients, and sample sizes varied from 40 to 306. The included studies comparing surgical and non-surgical management of intermittent exotropia and the study findings demonstrate that both management approaches can improve motor alignment and binocular function; however, the magnitude of success, sustainability, and timing differ between the approaches. Overall, both surgical and non-surgical treatments are effective for intermittent exotropia. However, outcomes vary depending on multiple factors such as age, treatment compliance, baseline deviation magnitude, and timing of intervention. In this context, treatment success depends on patient-specific characteristics and requires tailored approaches that account for them.

## Introduction and background

Intermittent exotropia (IXT) is among the most prevalent forms of childhood-onset strabismus and frequently persists into adulthood [1,2]. It is characterized by non-constant exodeviation that predominantly manifests at distance fixation and may progress over a variable period to near fixation [3]. According to Duane’s classification, IXT can be divided into four types: divergence excess, basic type, convergence insufficiency, and pseudo-divergence excess [4]. The global prevalence of IXT is estimated to range from 0.5% to 3.5% in population-based studies, while the annual incidence is 32.1 per 100,000 patients under the age of 19 [3,5].

Several theories have been proposed by different researchers to explain the underlying pathogenesis, including innervational imbalance [6], mechanical and anatomical factors [7], impaired fusional control [8], abnormalities in the accommodative convergence/accommodation (AC/A) ratio [9], and uncorrected refractive errors [10]. In adults, IXT may present with asthenopia, diplopia, reduced stereoacuity (3D vision), impaired binocular control, and psychosocial concerns [11,12].

Treatment of IXT is considered if caregivers observe that the eyes have been misaligned (exotropia) for a longer duration than they have been aligned and functioning together [13]. Currently, there is no universally accepted first-line treatment for IXT. Treatment of this condition can be surgical, non-surgical, or a combination of both. The main goal of surgical and non-surgical treatment of IXT is to preserve binocular vision and stereopsis, prevent further loss, and prevent amblyopia, which is not often found in patients with IXT [13,14]. Non-surgical treatments are considered for younger patients, patients who are unable to cooperate with examinations, with a small angle of exotropia, with good control, or patients who defer or delay surgical options [13]. Among the non-surgical approaches are occlusion therapy and the use of minus lenses to stimulate convergence, as well as orthoptic therapy, which consists of fusion exercises, that remain controversial due to ongoing debate regarding their efficacy and potential adverse effects on surgical outcomes. Orthoptic therapy may be useful only in cases of convergence insufficiency, as it can promote fixation and improve fusion.

Intermittent exotropia does not require immediate surgery; however, when the patient develops an increased frequency of strabismus, a larger basic deviation, or secondary convergence insufficiency, surgery is recommended to normalise the binocular function. This approach is the optimal management approach in cases of advanced IXT or when the non-surgical approach is not beneficial. The surgical approach includes two-muscle unilateral surgery, in which the medial rectus and lateral rectus muscles of one eye are resected to realign the visual axes; bilateral surgery, in which the previously mentioned approach is applied in both eyes; and one-muscle unilateral surgery, in which a single lateral rectus muscle is resected [15]. These approaches are customized for each patient based on their condition, associated conditions, age, and severity of the condition [16,17].

Currently, there is no clear evidence comparing the effectiveness of non-surgical approaches to surgical approaches in the treatment of IXT. Moreover, there is significant heterogeneity in the existing literature regarding the timing of surgery and whether it is more beneficial to operate before four years of age. The controversies in the existing literature extend to the incidence of the long-term recurrence of IXT, in which the majority of studies report that nearly 50% of patients require reoperation after long-term follow-up. Additionally, the heterogeneity in outcome definitions of the previous studies yielded a gap in the existing literature. This systematic review aims to assess and compare the effectiveness of surgical and non-surgical management of IXT. It also aims to explore the factors associated with lower or higher success rates among different treatment approaches of IXT.

## Review

Methods

Reporting Guidelines

This systematic review was conducted in accordance with the Preferred Reporting Items for Systematic Reviews and Meta-Analyses (PRISMA) guidelines [18]. A PRISMA flow diagram was used to document the study selection process.

Search Strategy

To conduct a comprehensive search for systematic reviews on the surgical and non-surgical management of IXT, an investigator searched a set of medical databases for randomised controlled trials (RCTs) and controlled clinical trials in English published within the last 10 years to focus on recent, methodologically robust studies reflecting current standards of care and modern outcome assessment in IXT. The last search was conducted on February 23, 2026. The search strategy employed a combination of keywords and Medical Subject Headings (MeSH) related to the PICO (Population, Intervention, Comparison, Outcome) framework (Table 1).

**Table 1:** PICO framework and search strategy PICO: Population, Intervention, Comparison, Outcome

Section	Component/Database	Description
PICO Framework	Population	Patients with intermittent exotropia
	Intervention	Surgical and non-surgical treatments
	Comparator	Not mandatory; may include observation, alternative interventions, or no treatment
	Outcomes	Primary: Motor alignment success; improvement in control of IXT. Secondary: Changes in angle of deviation, binocular function, recurrence rates, need for re-intervention, treatment-related adverse events, and age at onset
	Study Design	RCTs, secondary analysis of RCTs, post hoc study of RCTs
Search Strategy	PubMed	("Exotropia"[MeSH] OR intermittent exotropia OR intermittent exodeviation) AND ("Strabismus/surgery"[MeSH] OR surgery OR surgical OR orthoptic* OR occlusion OR patching OR observation OR nonsurgical OR non-surgical OR conservative) AND ("Randomized Controlled Trial"[Publication Type] OR randomized OR randomised OR randomly)
	ScienceDirect	("intermittent exotropia" OR "intermittent exodeviation") AND (surgery OR surgical OR orthoptic OR occlusion OR patching OR nonsurgical) AND ("randomized controlled trial")
	Cochrane Library	(intermittent exotropia OR intermittent exodeviation) AND (surgery OR surgical OR strabismus surgery OR orthoptic* OR occlusion OR patching OR observation OR nonsurgical OR conservative): ti,ab,kw

Keywords and search terms were combined using Boolean operators (AND/OR) to identify relevant studies. Filters were applied to include only English-language publications and studies with keywords appearing in the title or abstract. The search was limited to studies published within the last 10 years to ensure relevance to the research question.

Older RCTs may not reflect current clinical practice standards, surgical protocols, or outcome assessment tools. Therefore, we planned to include more recent trials, as they are more likely to follow updated methodological standards, thereby improving the overall quality of the evidence.

Study Selection and Inclusion and Exclusion Strategy for Study Selection

Studies with patients diagnosed with IXT and studies with a randomized controlled trial design, post hoc analyses of RCTs, and secondary analyses of RCTs were included. Studies published from 2016 to 2026 were included. Studies with a study design other than RCTs, studies that were not conducted in English, and studies with participants diagnosed with other types of exotropia were excluded. Review articles, systematic reviews, and meta-analyses were excluded. Two reviewers independently conducted the searches and screened articles for eligibility.

Data Extraction

Data extracted from each included study included study characteristics (design, setting, sample size, and follow-up), participant details (age, sex, and baseline clinical status), intervention and comparator information, and outcome measures such as motor alignment, exotropia control scores, stereoacuity, binocular function, recurrence, re-intervention, adverse events, compliance, and factors influencing treatment outcomes. Inter-reviewer agreement during screening and data extraction was not formally quantified using Cohen’s kappa; however, discrepancies were resolved through discussion and consensus, with arbitration by a third reviewer when required.

Quality Assessment

The quality of the studies included was evaluated using the Cochrane Risk of Bias 2 (RoB 2) tool for randomized trials [19]. Disagreements between reviewers were resolved through discussion to reach consensus or by involving a third, senior reviewer for settlement regarding randomized controlled trials.

Statistical Analysis

A quantitative meta-analysis was not performed because of substantial clinical and methodological heterogeneity among the included studies regarding intervention types, outcome definitions, follow-up durations, and success criteria. Therefore, findings were synthesized narratively by grouping studies according to intervention type and summarizing treatment effects across predefined outcomes.

Results 

Search Results

**Figure 1:**
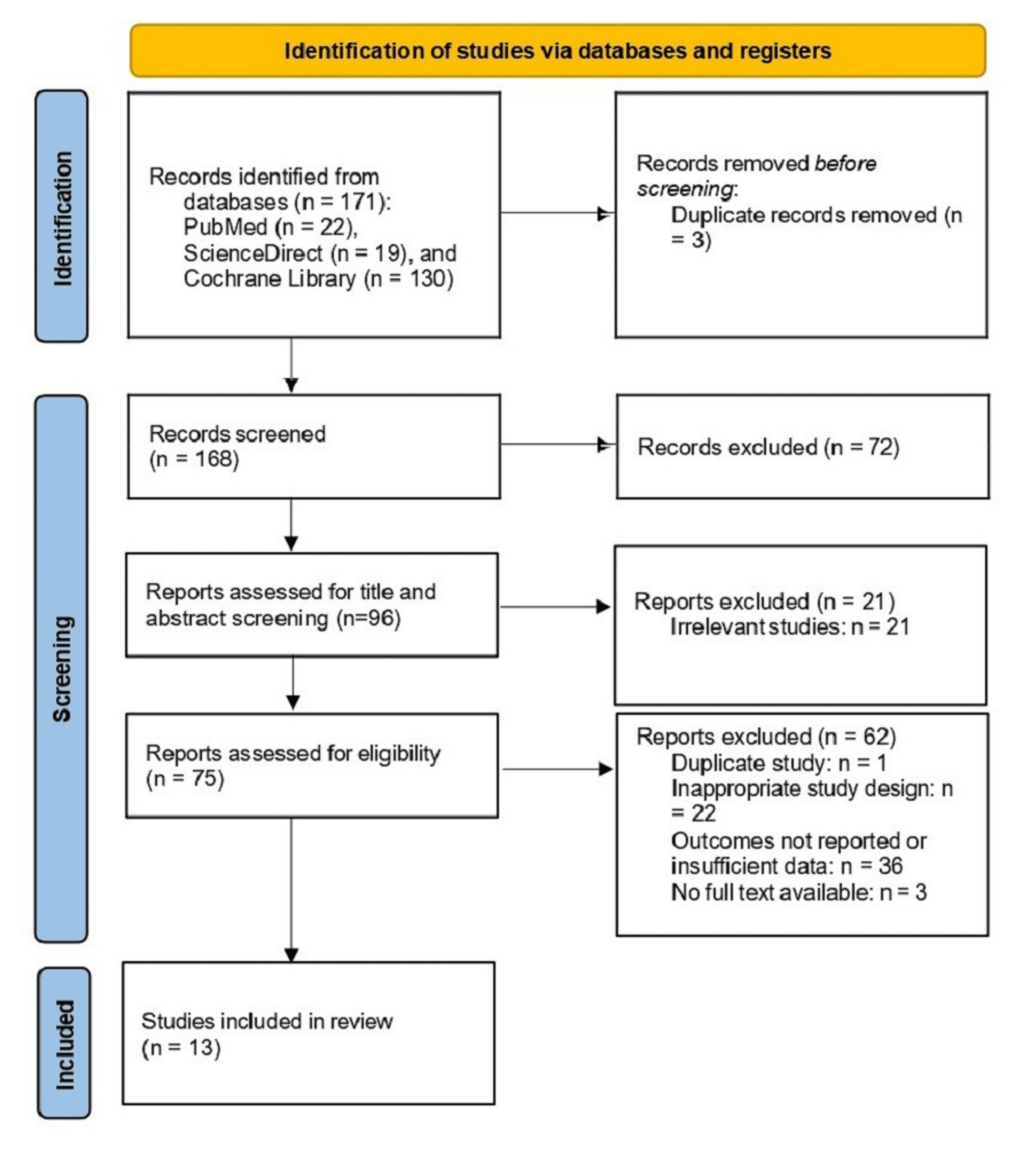
PRISMA flow diagram PRISMA: Preferred Reporting Items for Systematic reviews and Meta-Analyses

We executed the search methodologies outlined previously, resulting in the identification of a total of 171 citations, subsequently reduced to 168 following the removal of duplicates. Upon screening titles and abstracts, only 75 citations met the eligibility criteria for further consideration. Through full-text screening, this number was further refined to 13 articles [20-32] aligning with our inclusion and exclusion criteria. Figure 1 provides an in-depth depiction of the search strategy and screening process.

Quality Assessment Results

The risk-of-bias assessment of the included randomized controlled trials, post hoc studies of RCTs, and secondary analyses of RCTs was evaluated using RoB 2.0, which assessed variable methodological quality across 5 domains. In all five domains, including randomization, deviation from intended interventions, missing outcome data, and outcome measurement, many included randomized controlled trials showed a low risk of bias, indicating generally good methodological quality.

**Figure 2:**
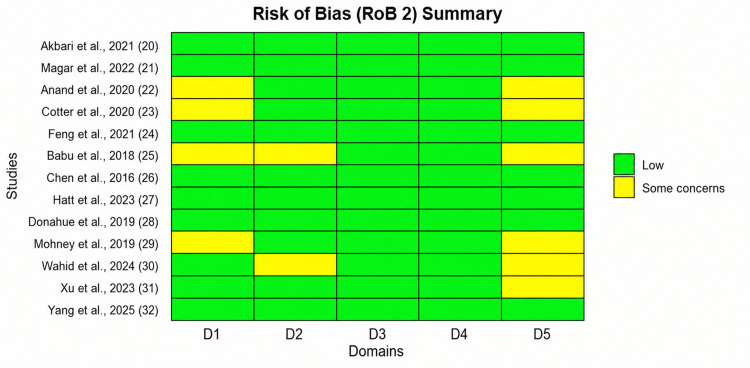
Cochrane risk-of-bias tool for randomized trials version 2 (RoB 2) Source: [20-32] D1: Bias arising from the randomization process; D2: Bias due to deviation from the intended intervention; D3: Bias due to missing outcome data; D4: Bias in measurement of the outcome; D5: Bias in the selection of the reported result

Nonetheless, the Anand et al. [22], Cotter et al. [23], Babu et al. [25], Mohney et al. [29], and Wahid et al. [30] studies showed some concerns, mainly due to potential limitations in the randomization process (D1) and possible selective reporting (D5) in the studies. Studies that were secondary analyses, post hoc reports, or had limited protocol transparency more frequently observed these concerns. No study was judged to be at high risk of bias in any domain, suggesting that the overall evidence base is relatively robust (Figure 2).

Baseline Characteristics of the Included Studies

Baseline study characteristics were summarized in Table 2. This systematic review comprised 13 studies in total, most of which were randomized controlled trials with a small number of observational studies. The majority of the research focused on pediatric populations; however, some included adult patients, and sample sizes varied from 40 to 306. The mean age ranged considerably, ranging from over 18 years in studies employing virtual reality-based therapies to about 2 years in observational populations. Intermittent exotropia was the most studied subtype (Table 2).

**Table 2:** Baseline characteristics of included studies LR: lateral rectus; MR: medial rectus; BLRc: bilateral lateral rectus recession; R&R: recession-resection; SERTA: The Study, Education and Research Trust Account; NIH: The National Institutes of Health; NSFC: National Natural Science Foundation of China; NRF: National Research Foundation; VR: virtual reality; CI: Convergence insufficiency; RCT: randomised controlled trial; SD: standard deviation; NR: not reported; I/C: intervention/comparator.

Study ID	Country	Design	Sample Size (I/C)	Age (mean ± SD)	% Male	Type of Exotropia	Intervention vs Comparator	Follow-up	Funding
Akbari et al., 2021 [20]	Iran	RCT	76 (35/41)	4.99 ± 1.33	38.20%	Intermittent	Patching vs observation	6 months	NR
Magar et al., 2022 [21]	Australia	RCT	141 (66/75)	6.8 ± 2.5	43%	Intermittent	Overminus lenses vs observation	6–15 months	SERTA
Anand et al., 2020 [22]	India	RCT	60 (30/30)	RP: 20.67 ± 9.22; RR: 19.13 ± 8.3	50%	Basic intermittent	LR recession + MR plication vs resection	12 months	NR
Cotter et al., 2020 [23]	Multicenter	Secondary analysis of RCT	97	24	40%	Intermittent	Observation (natural course)	3 years	NIH
Feng et al., 2021 [24]	China	RCT	60 (30/30)	4.75 ± 1.03	53.30%	Intermittent	Overminus + prism vs observation	12 months	NSFC
Babu et al., 2018 [25]	India	RCT	40 (20/20)	24.5 ± 4.3	Group A had 25% men, and Group B had 50% men	Intermittent	Adjustable vs non-adjustable sutures	6 months	NR
Chen et al., 2016 [26]	Multicenter	RCT	58 (27/31)	5.1 ± 1.1	38%	Intermittent	Overminus lenses vs observation	8 weeks	NIH
Hatt et al., 2023 [27]	Greece	Post hoc RCT	306 (142/164)	3–11 years	46%	Intermittent	Patching vs observation	6 months	NIH
Donahue et al., 2019 [28]	Multicenter	RCT	197 (101/96)	6.2 ± 2.0	BLRc group, 37%; R&R group: 40%	Basic intermittent	BLRc vs R&R	3 years	NR
Mohney et al., 2019 [29]	USA	Secondary analysis of RCT	183	6.1 ± 2.0	37%	Intermittent	Observation	3 years	NIH
Wahid et al., 2024 [30]	Egypt	RCT	40 (20/20)	Control:10.2 ± 2.58; study group:10.75 ± 2.93	55%	Post-surgical intermittent	Physiotherapy vs usual care	3 months	NR
Xu et al., 2023 [31]	China	RCT	136 (68/68)	10.4 ± 2.5	51.40%	Basic intermittent	Orthoptic therapy vs observation	12 months	NSFC
Yang et al., 2025 [32]	Korea	RCT	62 (31/31)	Exercise group: 18.2 ± 5.6; control group: 18.0 ± 5.3	Exercise group: 48.4%; control group:61.3%)	Basic/CI type	VR therapy vs placebo	8 weeks	NRF

The included studies comparing surgical and non-surgical management of IXT and the study findings demonstrate that both management approaches can improve motor alignment and binocular function; however, the magnitude of success, sustainability, and timing differ between the approaches. Surgical interventions, such as recession-resection, bilateral lateral rectus recession, and adjustable suture techniques, consistently show higher initial motor alignment success rates. Based on the included studies (approximately 60%-90%) [22,25], there are substantial reductions in the angle of deviation (of up to 16-18 PD in Donahue et al. [28]. Additionally, stereoacuity and binocular function tend to improve faster after surgery, although overcorrection and early postoperative recurrence remain common challenges. Factors such as preoperative deviation magnitude and type of surgical technique influence both short-term alignment and the need for secondary procedures. In contrast to that, non-surgical interventions, such as patching, overminus lenses, orthoptic therapy, and observation, demonstrated moderate but considerable improvements, particularly in control scores and near/far deviation reduction. Orthoptic exercises improved fusional control, while overminus lenses and combined prism therapy produced notable short-term improvements in distance and near control. Recurrence was common (34% vs 26% in Donahue et al. [28]), and re‑operation rates were higher in some surgical groups. Observational studies show that deterioration over time is rather minimal, particularly in mild cases, indicating that not all patients may require prompt surgical intervention (Table 3).

**Table 3:** Study outcomes of surgical and non-surgical management of intermittent exotropia (IXT) BLRc: bilateral lateral rectus recession; R&R: recession-resection; NR: not reported; PD: prism diopter; AEs: adverse events; OML: Overminus Lens; RP: Recession-Plication; PACT: Prism and Alternate Cover Test; AS: Adjustable Suture; NAS: Non-Adjustable Suture; PT: points; IXTQ: Intermittent Exotropia Questionnaire; VR: virtual reality; NCS: Newcastle Control Score.

Study	Follow-up	Intervention/Treatment Approach	Motor Alignment Success (n/N, %)	Definition of Success	Control Improvement (Distance/Near)	Stereoacuity Outcome	Angle of Deviation Change (PD)	Binocular Function/Fusion	Recurrence Rate (n/N, %)	Time to Recurrence	Re-intervention (n/N, %)	Adverse Events	Quality of Life/Patient-Reported Outcome	Factors Influencing Success
Akbari et al., 2021 [20]	6 months	Patching vs. Observation	NR	Deviation control 3-point & 6-point scale	At far: 3 months p=0.033, 6 months p=0.16; At near: 3- & 6-months p=0.011	Greater improvement in patching group from baseline to 3 & 6 months (p=0.006)	NR	NR	NR	NR	NR	No significant AEs	NR	Age not specifically analyzed
Magar et al., 2022 [21]	15 months	Overminus Lens (OML) vs. Observation	NR	Improvement in IXT control score	OML: Distance 1.6 ± 1.3, Near 0.6 ± 0.7 vs Observation: Distance 3.7 ± 1.0, Near 1.9 ± 1.0	Significant improvement in OML group (p<0.001 distance, p=0.001 near)	Distance: OML -6.9 ±7.2 PD, Near -6.2 ±8.6 PD vs Obs 0.0 ±4.2 PD	NR	NR	NR	NR	Blurred vision in 4%; most participants asymptomatic	NR	Compliance affected success
Anand et al., 2020 [22]	12 months	Recession-Plication (RP) vs. Recession-Resection (RR)	RP: 66.7%, RR: 76.7%	Post-patch deviation 10 PD exo → <5 PD eso	NR	Improvement in 11 (RP) vs 12 (RR)	Distance deviation: RP 44.7 → 10.1 PD, RR 43.2 → 9.5 PD	Improved	NR	NR	NR	No complications, no esotropia	NR	Preoperative deviation magnitude not predictive
Cotter et al., 2020 [23]	6–12 months	Orthoptic Therapy vs Control	83% vs 60.3%	Exotropia <10 PD, Esotropia <5 PD	Distance control: 86.4% vs 67.2%, Near: 91.5% vs 67.2%	NR	Exodeviation drift: -3.9 ±6.6 PD vs -5.1 ±6.2 PD	Fusional exotropia control improved	NR	NR	NR	NR	-	-
Feng et al., 2021 [24]	12 months	Overminus + Prism vs Observation	NR	Newcastle Control Score ≤3	Treatment group 71.4% NCS ≤3 vs Obs 6.89%	Near stereoacuity improved: 1.91 vs 2.16 log arcsec	Near PACT reduced: 16.25 ±7.03 PD vs 26.72 ±7.41 PD	Improved binocular function	NR	NR	NR	No myopia at 12 months	-	-
Babu et al., 2018 [25]	12 weeks	Adjustable Suture (AS) vs Non-Adjustable Suture (NAS)	AS 90%, NAS 85%	<10 PD deviation	NR	Near stereoacuity: AS 48″, NAS 52″	Postop distance: AS 6.2 PD, NAS 5.6 PD	Acceptable binocular status: AS 35%, NAS 60%	NR	NR	20% AS required additional recession	Minimal scar; 2 persistent red eyes	-	-
Chen et al., 2016 [26]	8 weeks	Overminus vs Observation	Distance control ≥1 pt: 59% vs 39%	≥1 pt improvement in distance control	Distance control score: Overminus 2.0 vs Obs 2.8; Change: -1.2 vs -0.4	No significant difference	Near PACT: Overminus 12.8 Δ vs Obs 16.9 Δ	NR	NR	NR	NR	Headache, eye strain, blur, rare	NR	Suture adjustability not significantly associated with success
Hatt et al., 2023 [27]	3 & 6 months	Patching vs Observation	28% vs 17%	≥2-point improvement in control	Distance control: 3m mean diff 0.4 pts, 6m 0.3 pts	NR	Near deviation improved 2.3° vs 0.2°	NR	NR	NR	NR	-	NR	Early intervention improved distance and near control
Donahue et al., 2019 [28]	3 years	BLRc vs RR	Suboptimal outcome: 46% vs 37%	Suboptimal = exotropia ≥10 PD, eso ≥6 PD, stereo loss ≥2 octaves, reoperation	Distance control improvement: 2.3 vs 2.5 pts	Stereoacuity: no significant change	Distance PACT reduction: 16 PD vs 18 PD	NR	Exotropia ≥10 PD: 34% vs 26%	NR	Reoperation: 10% vs 5%	Minor postop cysts/edema	IXTQ scores not different	Timing and type of surgery did not significantly affect long-term control
Moheny et al., 2019 [29]	3 years	Observation (untreated)	<1% deterioration	Deterioration: constant exotropia ≥10 PD or stereoacuity drop >2 octaves	Distance exotropia control improved 0.6 pts	Near & distance stereo improved 0.14 log arcsec	Distance exodeviation improved 2.2 PD	NR	NR	NR	10% received treatment, 3% surgery	NR	Natural course shows low deterioration in mild IXT	-
Wahid et al., 2024 [30]	12 weeks	Physical Therapy Exercises	NR	Improvement in exotropia control (near & far)	NR	NR	NR	NR	NR	NR	NR	NR	NR	Structured exercises improved control independent of surgery
Xu et al., 2023 [31]	6 months	Surgical/Non-surgical	68/85 (80%)	<10 PD	Distance control of IXT significantly improved in the treatment group (p=0.004)	Improved	-3.5 PD	Improved	10/85 (12%)	3 months	5/85 (6%)	None reported	Improved	Age at onset, Timing of surgery
Yang et al., 2025 [32]	12 weeks	VR Therapy	25/30 (83%)	<8 PD	Patients in the Virtual Reality (VR) therapy group showed a significant improvement (p=0.032)	Improved	-2.0 PD	Improved	3/30 (10%)	2 months	1/30 (3%)	None reported	Improved	VR compliance, Baseline deviation

Factors influencing treatment success include age at onset, treatment compliance, preoperative deviation magnitude, and timing of intervention [20-22,28]. Younger patients and those receiving early, structured non-surgical therapy often achieve better control scores, whereas higher preoperative deviation or delayed surgical intervention may increase the risk of residual exotropia or reoperation. Long-term outcomes highlight that while surgery offers higher initial success, sustained binocular function and recurrence rates can be influenced by postoperative management and individual patient characteristics (Table 3).

A detailed summary of evidence on factors influencing treatment outcomes across included studies is presented in Table 4.

**Table 4:** Study-level characteristics and potential sources of outcome variability among included studies

Study	Mean Age (years)	Intervention	Follow-up Duration	Treatment Compliance Assessed	Baseline Deviation Considered	Timing of Intervention Evaluated	Potential Sources of Outcome Variability Reported or Evaluated
Akbari et al., 2021 [20]	4.99 ± 1.33	Alternate part-time patching vs observation	6 months	Yes	No	No	Patching improved deviation control and stereoacuity changes compared with observation. Compliance was monitored throughout follow-up, but no independent predictors of treatment success were formally identified.
Magar et al., 2022 [21]	6.8 ± 2.5	Overminus lens therapy vs observation	6–15 months	Yes	Yes	Yes	Baseline control score, deviation magnitude, accommodative convergence (AC)/accommodation (A) ratio, refractive error, and treatment compliance were evaluated as potential determinants of treatment response.
Anand et al., 2020 [22]	19–21	Recession–Resection (RR) vs Recession–Plication (RP)	12 months	Not reported	Yes	Yes	Baseline deviation, binocular function, and control status were assessed as potential contributors to surgical outcomes; AC/A ratio was evaluated but was not associated with success.
Cotter et al., 2020 [23]	2–10*	Observation (natural history)	3 years	Not applicable	Yes	Yes	Baseline control score, deviation magnitude, and binocular status were evaluated as potential factors associated with progression or deterioration.
Feng et al., 2021 [24]	4.75 ± 1.03	Overminus lenses plus prism vs observation	12 months	No	Yes	Yes	Baseline control score and deviation magnitude were evaluated as potential determinants of treatment response.
Chen et al., 2016 [26]	5.1 ± 1.1	Overminus spectacles vs observation	8 weeks	Yes	Yes	Yes	Baseline severity, treatment compliance, and previous treatment history were evaluated as potential contributors to outcome variability.
Hatt et al., 2023 [27]	3–<11 years	Part-time patching vs observation	3–6 months	No	Yes	Yes	Baseline control score and deviation magnitude were evaluated as potential contributors to treatment response; regression-to-the-mean effects were discussed.
Donahue et al., 2019 [28]	6.2 ± 2.0	Bilateral lateral rectus recession (BLRc) vs recession–resection (R&R)	3 years	Yes	Yes	Yes	Baseline control score, deviation magnitude, stereopsis, reoperation status, and surgical approach were evaluated as potential contributors to long-term outcomes.
Mohney et al., 2019 [29]	6.1 ± 2.0	Observation	3 years	Not applicable	Yes	Yes	Baseline control score, deviation magnitude, and stereopsis were evaluated as potential predictors of deterioration and subsequent treatment requirement.
Wahid et al., 2024 [30]	10.75 ± 2.93	Postoperative physiotherapy vs standard care	3 months	Partial	Yes	Yes	Baseline control status, adherence to therapy, and treatment intensity may have contributed to variability in outcomes.
Xu et al., 2023 [31]	10.4 ± 2.5	Orthoptic therapy vs control	6–12 months	Yes	Yes	Yes	Treatment compliance, baseline fusional status, and convergence reserve were evaluated as important determinants of therapeutic response.
Yang et al., 2025 [32]	18.1 ± 5.4	Virtual reality-based orthoptic therapy vs placebo virtual reality (VR)	4–8 weeks	Yes	Yes	Yes	Treatment adherence (VR usage), baseline control status, and intervention duration were evaluated as potential contributors to treatment response and sustainability of effects.

Discussion

This systematic review aimed to evaluate and compare the effectiveness of surgical and non-surgical interventions for the treatment of IXT. It included 14 studies, with sample sizes ranging from 40 to 306 participants. The mean ages of participants ranged from 2 to 24 years. Pediatric patients were predominant, as IXT is more prevalent among children, given that their binocular vision system is still developing before the age of six. Both surgical and non-surgical approaches were reported to be effective in improving motor alignment and binocular visual function. However, they differ in the magnitude of success, sustainability, and timing.

Comparison of Surgical vs Non-surgical Outcomes

Our findings indicate that surgical interventions result in significantly higher initial motor alignment success rates, typically ranging from 66% to 91% [22,33]. The direct mechanical correction of the imbalance in the extraocular muscle, in techniques such as bilateral lateral rectus recession (BLRc) and unilateral recession-resection (R&R), demonstrated substantial reductions in angle deviations, with mean reductions up to 16-18 prism diopters (PD) [28]. In contrast to non-surgical interventions, which rely on neurosensory adaptation and behavioral compensation, yielding more gradual improvements in alignment. Moreover, plication and resection techniques resulted in significant improvements in alignment, with no statistically significant differences between the techniques [22]. Notably, both adjustable and non-adjustable suture techniques also showed high success rates (90% vs 85%) with no significant difference, indicating that suture adjustability may not have a significant impact on alignment outcomes [25].

Despite rapid and substantial alignment correction during surgical interventions, surgical management is associated with higher recurrence and reoperation rates. For instance, cumulative suboptimal outcomes were reported in 46% of BLRc cases and 37% of R&R cases at 3 years of age [28]. Moreover, early overcorrection was particularly observed in BLRc procedures [33]. In contrast, non-surgical interventions demonstrated moderate yet clinically significant improvements in both distance and near control scores and in binocular function compared with observation. Overminus therapy reduced the baseline deviation angle by a range of 6 to 7 PD and significantly improved stereoacuity, as reported by Magar et al. (2022) [21]. Moreover, the combination of overminus and prism therapies further enhanced outcomes, improving both stereopsis and deviation control [24].

In terms of adverse events, non-surgical interventions, such as overminus lenses, were associated with transient symptoms, including blurred vision and eye strain [21,26]. On the contrary, surgical groups reported minor postoperative complications, such as cysts or edema [28]. No significant or severe complications were consistently reported. The relatively low risk profile supports the safety of both approaches. Based on current evidence, the quality-of-life (QOL) outcomes were infrequently reported, with no significant differences between groups. Donahue et al. (2019) assessed the QOL of patients with IXT using the Intermittent Exotropia Questionnaire (IXTQ), which consisted of 3 components that assessed IXT-related health-related QOL (HRQOL) as reported by children, the child’s HRQOL as perceived by the parents, and the parents’ own HRQOL on three subscales on psychosocial, functional, and surgical domains [28]. They reported no significant differences in IXTQ scores between surgical techniques [28]. This may be attributed to the fact that the QOL is multifactorial, influenced not only by alignment but also by psychosocial adaptation, symptom perception, and patient expectations. Additionally, short follow-up durations in many studies may have hindered the detection of significant differences.

Factors Influencing Treatment Success

Age was reported as one of the factors influencing treatment outcomes. Younger children receiving early non-surgical therapies, such as patching and orthoptic exercises, achieved more pronounced improvements in near- and distance-control scores [20,23,27]. This is consistent with Akbari et al. (2021), who reported that part-time patching in children aged 3-6 years led to significant improvements in near control at 3 months (p=0.011) and short-term gains in distance control (p=0.033) [20]. Similarly, Hatt et al. (2023) observed better early control among children aged 3-11 years receiving patching therapy, highlighting the benefit of early structured interventions [27]. In contrast, studies involving adults and older pediatric populations, such as that by Yang et al. (2025), which included participants with a mean age of 18.2 years and used virtual reality therapy, reported smaller improvements and a stronger influence of treatment compliance [32].

Long-Term Follow-Up and Recurrence Rate

 Although both approaches showed remarkable success in improving motor alignment and binocular function, especially the surgical approach, the recurrence rate remains high. Kopmann et al. reported that after 4 days to 20 years of follow-up after surgery, out of 176 patients, 40 patients were still in the range of surgical success, 133 patients had exotropia > 10 PD, and 65 patients underwent reoperation [33]. A total of 8.5% had their reoperation within one year after the first surgery, 52.9% within five years. These findings are in line with our results, in which recurrences were common, and reoperations were high. This underscores the importance of conducting studies with long-term follow-up to obtain a more accurate evaluation of the success rates of surgical and non-surgical approaches to assess which approach is more effective and results in fewer recurrences and less need for reoperations.

Overall, treatment outcomes vary depending on multiple factors, including age at onset, treatment compliance, baseline deviation magnitude, and timing of intervention. On this account, younger patients and those with better adherence to treatment demonstrated improved outcomes, whereas those with larger baseline deviations were associated with an increased risk of recurrence or the need for surgical intervention. These findings highlight that treatment success is based on patient-specific characteristics and disease severity, which influence both motor and sensory adaptation.

Strengths and Limitations

This review has several strengths, including the inclusion of multiple RTCs with diverse interventions ranging from conventional surgery to modern approaches such as virtual reality (VR) therapy. The long-term observational studies also help provide a comprehensive evaluation of current surgical and non-surgical approaches. However, the study is limited by the variability in study designs, outcome measures, and follow-up durations, limiting direct comparisons. A major limitation of the included evidence is the heterogeneity in outcome definitions, where “treatment success” varied widely across studies, including prism diopter thresholds, control scores, stereoacuity changes, and composite outcomes. This variability limits direct comparability and reinforces the need for standardized outcome reporting in intermittent exotropia research. The lack of standardization in measuring outcome success, such as deviation thresholds and control scores, complicates data analysis. Moreover, the short follow-up periods, particularly for non-surgical interventions, limit the assessment of long-term sustainability. The absence of a quantitative meta-analysis limits the ability to generate pooled effect estimates and formally assess statistical heterogeneity.

Clinical Implications and Recommendations

While both surgical and non-surgical interventions are effective in the management of IXT, surgical treatment has shown rapid motor alignment, and non-surgical therapies offer non-invasive alternatives for mild-to-moderate cases. Future research should focus on comparing long-term effectiveness, standardizing outcome measures, in addition to evaluating the effectiveness of combined treatment strategies and optimizing the timing of intervention to improve both motor and sensory outcomes. Future research should also focus on integrating novel technologies, such as botulinum toxin injection into the lateral rectus muscles to manage intermittent exotropia, which has shown promising results in the short term.

## Conclusions

While both surgical and non-surgical interventions were reported to be effective for the treatment of IXT, surgical approaches provide higher initial alignment success, and non-surgical therapies offer moderate long-term improvements in control. Given that treatment outcomes are influenced by several factors, including age, baseline angle deviation, disease severity, and compliance, personalized treatments that account for patient-specific characteristics are required, with further research to inform evidence-based practice.
